# Detection of circulating type 3 vaccine-derived polioviruses in French Guiana, May to August 2024

**DOI:** 10.2807/1560-7917.ES.2024.29.45.2400705

**Published:** 2024-11-07

**Authors:** Stéphanie Raffestin, Ambre Tinard, Antoine Enfissi, Marie-Line Joffret, Timothée Lichtenstein, Sourakhata Tirera, Laura Zanetti, Marie Barrau, Francky Mubenga, Adrien Ortelli, Christophe N Peyrefitte, Anne Lavergne, Dominique Rousset, Maël Bessaud

**Affiliations:** 1 Institut Pasteur de la Guyane, Laboratoire hygiène et environnement, Cayenne, France; 2 Consortium OBEPINE, Paris, France; 3 Institut Pasteur, Université Paris Cité, CNRS UMR 3569, Signalisation antivirale, Paris, France; 4 Laboratoire associé au Centre national de référence entérovirus/paréchovirus, Paris, France; 5 Centre collaborateur de l’OMS, Épidémiologie et macro-évolution des poliovirus and entérovirus non-polio, Paris, France; 6 Institut Pasteur de la Guyane, Laboratoire de virologie, Cayenne, France; 7 Institut Pasteur de la Guyane, Laboratoire des interactions virus-hôtes, Cayenne, France; 8 Santé publique France, Direction des maladies infectieuses, Saint-Maurice, France; 9 Santé publique France, Direction des régions, Cellules Guyane, Cayenne, France; 10 Agence régionale de santé de Guyane, Direction de la santé publique Cayenne, France; 11 Institut Pasteur de la Guyane, Cayenne, France

**Keywords:** poliovirus, poliomyelitis, vaccine-derived poliovirus, oral polio vaccine, environmental surveillance

## Abstract

Circulating type 3 vaccine-derived polioviruses (cVDPV3s) were detected in three wastewater samples collected in French Guiana from May through August 2024. As the oral polio vaccine is not used in French Guiana, this event involved an import either of cVDPV3s themselves or of a vaccine strain from which the cVDPV3s emerged in French Guiana. This highlights the importance of environmental surveillance for the detection of silent poliovirus circulation. Eliminating any pockets of cVDPVs is crucial for the polio eradication programme.

We report the genetic characterisation of type 3 vaccine-derived polioviruses (VDPV3) found in wastewater samples collected in Cayenne’s greater metropolitan area and in Saint-Georges-de-l’Oyapock. These viruses were detected in the framework of a collaborative project that aims to characterise enteroviruses (both polioviruses and non-polio enteroviruses) that circulate in French Guiana. As the VDPV3s were genetically linked and were sampled in May, June and August 2024, they met the criteria that define circulating VDPVs (cVDPVs). Detecting cVDPVs is crucial for the Global Polio Eradication Initiative since they reveal immunisation gaps that allow polio vaccine strains to circulate.

## Situation in French Guiana

**Figure 1:**
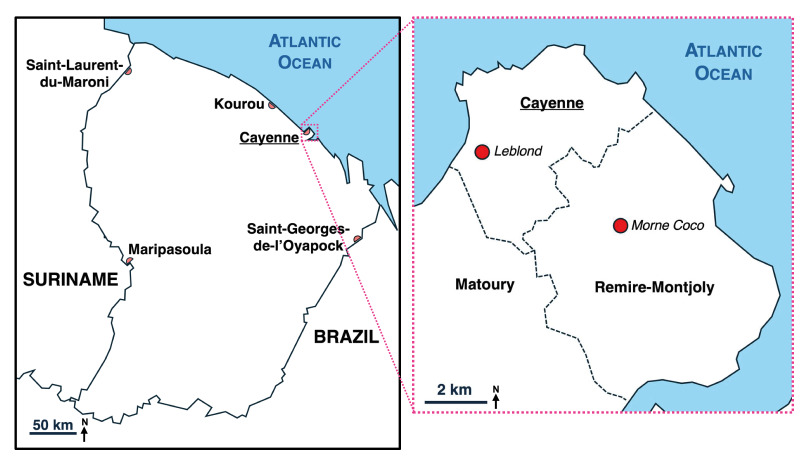
Map of French Guiana and greater metropolitan area of Cayenne

French Guiana is a French overseas department located in South America bordering Suriname and Brazil that covers around 83,000 km^2^ (Figure 1). The department has ca 300,000 inhabitants, most of which live in Cayenne, the capital city, and its greater metropolitan area (ca 150,000 inhabitants) and in Saint-Laurent-du-Maroni (ca 50,000 inhabitants). As a French department, French Guiana follows the French vaccination schedule. Since 1982, the inactivated polio vaccine (IPV) has been used exclusively for routine immunisation. From 1964 through 2018, this vaccination was included in the list of mandatory vaccines for children up to 12 years of age. Since 2018, polio vaccination has been compulsory for all children born after 1 January 2018 before entering a childcare centre or any collective childhood institutions [1]. While polio immunisation coverage in mainland France is above 95% in most regions, the vaccination coverage in French Guiana is difficult to estimate with accuracy. The last case of poliomyelitis in French Guiana was reported in 1986 [2].

On the left map, the main cities are indicated by orange half circles. On the right map, red circles indicate the location of the two wastewater treatment facilities where positive samples were collected in the greater metropolitan area of Cayenne.

## Detection of polioviruses in wastewater samples by virus isolation

Routine poliovirus (PV) environmental surveillance (ES) is no longer carried out in France. Search for PVs in wastewater samples was conducted in Paris and its suburbs from 1973 through 2018 but was stopped because of the low number of positive samples (3 from 2000 through 2018) [3]. 

In the framework of a collaborative research project that aims to build up ES capacities in France, the Global Polio Specialised Laboratory hosted by the Institut Pasteur, Paris, received leftovers (ca 250 mL) of 19 wastewater samples initially received by the Institut Pasteur de la Guyane, Cayenne, for routine sanitary controls of water. These samples were collected from June through August 2024 in different wastewater treatment facilities in the Cayenne’s metropolitan area, Kourou and Saint-Laurent-du-Maroni. Leftover raw wastewater samples were sent to the Institut Pasteur, Paris and then concentrated as previously described [4]. In compliance with the World Health Organization (WHO) guidelines for PV environmental surveillance [5], the concentrates were used to inoculate two cell lines: the RD cell line (human rhabdomyosarcoma), which is highly sensitive to PVs but also amplifies other enteroviruses, and the transgenic murine L20B cell line, which is more specific to PVs [6]. RD-positive supernatants are cross passaged on L20B cells. The supernatants of all L20B cultures in which cytopathic effects are observed are molecularly screened for the presence of PV. Two samples collected in the Cayenne metropolitan area (wastewater treatment facilities Leblond and Morne Coco, Figure 1) gave positive results in L20B cells (Table), while the other 17 samples were positive in RD cells only (8 samples) or negative in both (9 samples). 

**Table:** Wastewater samples containing type 3 vaccine-derived polioviruses, French Guiana, May–August 2024 (n = 3)

Sample	Location	Collection date	Isolation in L20B cells^a^	Direct amplification^a^
ENV-GUF-SGO-SGO-2405–002	Saint-Georges-de-l’Oyapock	15 May 2024	Not tested	Positive (PQ467719)
ENV-GUF-CAY-LEB-2406–001	Cayenne	26 Jun 2024	Positive (PQ467717)	Not tested
ENV-GUF-CAY-MCO-2408–016	Remire-Montjoly	6 Aug 2024	Positive (PQ467716)	Positive (PQ467718)

^a^ GenBank accession numbers of the corresponding genetic sequences are provided.

Following the Global Polio Laboratory Network (GPLN) algorithm, the L20B-positive supernatants were molecularly screened with the Intratypic differentiation assay version 5.0 [7], which revealed the presence of PVs. Their VP1-encoding genomic region was amplified with the Y7/Q8 primer pair [8] and sequenced by the Sanger technique for genetic characterisation.

## Detection of polioviruses in wastewater samples by molecular screening

In response to the initial PV detection, the Institut Pasteur de la Guyane performed retrospective and prospective molecular screening on 61 wastewater samples collected from May through August 2024 in nine wastewater treatment facilities in Saint-Laurent-du-Maroni, Kourou, Cayenne’s greater metropolitan area and Saint-Georges-de-l’Oyapock. Briefly, wastewater samples were homogenised, and 11 mL were ultracentrifuged at 200,000 x g for 1 h at 4 °C. Pellets were resuspended in 500 µL of phosphate-buffered saline and then lysed using 2 mL of NucliSENS lysis buffer (bioMérieux). After clarification by centrifugation at 30,000 x g for 5 min, nucleic acids were extracted from the supernatants using the EMAG platform (bioMérieux) and inhibitors were removed using the OneStep-96 PCR Inhibitor Removal kit (Ozyme). The RNA extracts were screened by real-time RT-PCR using a Sabin 3-specific assay previously described [7]. 

Eleven of 61 samples gave positive results, all with late quantification cycle (Cq) values (> 37.0). Seven samples, which featured Cq values above 40.0, were not analysed further. The four positive samples that featured the lowest Cq values (37.0–39.0) were used to attempt an amplification covering the VP1-encoding region; three samples came from Cayenne’s greater metropolitan area and one Saint-Georges-de-l’Oyapock. After RT-PCR with the Y7/Q8 primer pair, semi-nested PCRs were performed with Sabin-3 specific primers [7] generating overlapping amplicons that span the VP1-encoding region. This strategy was successful for two samples (Table). The corresponding amplicons were sequenced by nanopore sequencing (Oxford Nanopore Technologies). One sample from which an amplicon was obtained after direct amplification was also positive in cell cultures (sample ENV-GUF-CAY-MCO-2408–016; Table); the other (sample ENV-GUF-SGO-SGO-2405–002) was not tested by cell culture inoculation because of an insufficient leftover volume.

## Genetic characterisation of the polioviruses

**Figure 2:**
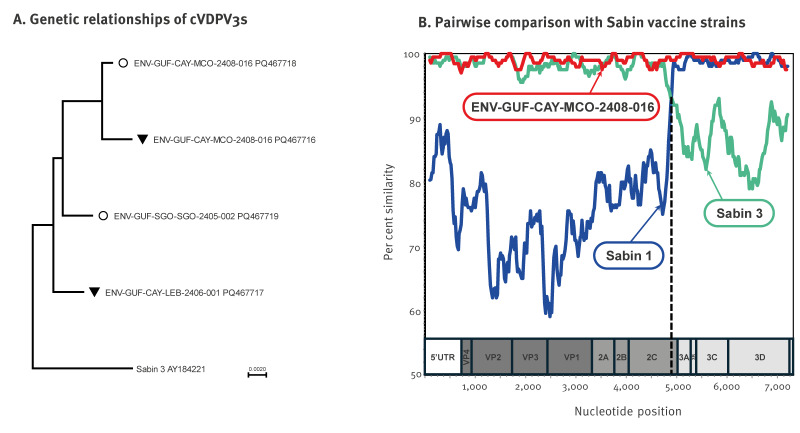
Genetic analysis of detected type 3 vaccine-derived polioviruses, French Guiana, June–August 2024 (n = 4)

Overall, four VP1 sequences (Figure 2A) were obtained (two through cell culture, two through direct amplification). The VP1 sequences obtained from sample ENV-GUF-CAY-MCO-2408–016 by both techniques differed from each other by four nucleotides, which is not surprising since wastewater samples may contain multiple PVs. The four VP1 sequences were derived from the type 3 vaccine strain, so-called Sabin 3, from which they differed by 15–20 nucleotides, which is beyond the threshold (10 differences) used to discriminate Sabin 3-like strains from VDPV3s [9]. Eleven nucleotide changes were shared by the four VDPV3s, which indicated a common emergence. Based on this genetic linkage and considering evidence of multiple detections between May and August 2024 in distinct collection sites, these VDPV3s were classified as circulating VDPV3s (cVDPV3s), according to the GPLN guidelines [9]. The cVDPV3s detected in French Guiana did not have any genetic linkage with PV3 strains previously submitted to the GPLN. Therefore, the origin of importation could not be determined.

A. Genetic relationships between the detected type 3 vaccine-derived polioviruses based on the VP1-encoding region. The evolutionary history was inferred in MEGA X [27] by using the Maximum Likelihood method and Tamura-Nei model. The tree is drawn to scale, with branch lengths measured in the number of substitutions per site. Triangles indicate the virus isolates while the open circles indicate the sequence obtained by direct sequencing. The sequence of the Sabin 3 vaccine strain was used to root the tree.

B. Pairwise comparison of the genome of the ENV-GUF-CAY-LEB-2406–001 isolate with those of ENV-GUF-CAY-MCO-2408–016 isolate, Sabin 1 and 3 vaccine strains. The dashed line indicates the Sabin3/Sabin 1 breakpoint.

Whole genome sequencing of the two isolates revealed that they differed from each other by 73 nucleotides. They displayed the same recombinant genetic structure between Sabin 3 and Sabin 1 vaccine strains. The breakpoint was located within the 2C region (Figure 2B), which is a Sabin 3 recombination hotspot [10]. The three major determinants of the Sabin 3 attenuated phenotype (nucleotide positions 472, 2,034 and 2,493) had reverted, as had the Sabin 1 attenuation determinant at nucleotide position 6,203 [11]. Therefore, the cVDPV3s have likely recovered a neurovirulent phenotype.

## Response to the detection of VDPV3s

The detection of cVDPV3s was reported on 2 August 2024 to Santé publique France (the French national public health agency), to the French health authorities and to the GPLN. Although no suspected poliomyelitis cases have been reported in French Guiana, a rapid response has been launched to strengthen polio immunisation and surveillance. In October 2024, a catch-up vaccination campaign employing IPV started in the Cayenne’s greater metropolitan area and in Saint-Georges-de-l’Oyapock, where cVDPV3s were detected [12]. In line with the French vaccination schedule, it mainly targets children aged < 6 years and adolescents aged 11 to 13 years old. Healthcare professionals have been sensitised to check patients’ vaccination records and to report any potential acute flaccid paralysis. In addition, a new routine ES programme has been funded by the Agence régionale de santé de Guyane for 1 year, based on samples regularly collected in different wastewater treatment facilities of French Guiana.

## Discussion

The Global Polio Eradication Initiative has led to the eradication of wild PVs of serotypes 2 and 3, while wild PV1s still circulate in two countries only, Pakistan and Afghanistan [13]. Since 2016, most poliomyelitis cases have been due to cVDPVs, which can emerge when vaccination gaps allow vaccine strains to circulate and revert [14]. In the last 5 years, most cVDPV outbreaks have been due to cVDPV2s, mainly in sub-Saharan Africa [15]. cVDPV1 outbreaks were also reported in the Democratic Republic of Congo, Mozambique and Madagascar [16,17]. cVDPV3 outbreaks are rare and the last event involving such viruses took place in 2021–22 in Israel [18]. Therefore, the detection of cVDPV3 in French Guiana constitutes an unexpected event, especially since the oral polio vaccine (OPV) is not used in this territory. The lack of data on actual vaccination coverage among the population of French Guiana and the presence of a relatively high number of immigrants whose vaccination status is unknown do not allow an estimate of the proportion of people likely to be affected by poliomyelitis among those residing in the territory.

Based on the PV estimated mutation rate in the VP1-encoding region (ca 1% per year [19]), the cVDPV3s found in French Guiana would have circulated for 1–2 years. Their geographic origin is unknown. Cross-border movements of people are common between French Guiana and its two bordering countries, Suriname and Brazil, both of which use OPV. Nonetheless, they could originate from any country using OPV since French Guiana has a high number of immigrants, especially from the Caribbean but also more recent asylum seekers from the Maghreb and Middle East [20,21]. The current data did not allow the determination of whether a local circulation has been established in French Guiana or whether the cVDPV3s were repeatedly imported from their region of origin. cVDPVs can circulate in countries with high vaccine coverage because of regional disparities and pockets of under-immunised individuals, as it was recently observed in the United Kingdom, Israel and the United States [4,22,23]. Moreover, because it does not induce a strong mucosal immunity, IPV is not an effective barrier to prevent PV circulation [24], as demonstrated by the 1-year circulation of a wild PV strain in Israel despite a vaccine coverage higher than 95% [25]. 

These past events and the detection of VDPV3s in French Guiana highlight the interest of ES for the detection of silent circulation of PVs. Although IPV is part of vaccination schedules in South America, ES has not been widely implemented in this region and, thus, widespread of PVs could occur and remain undetected in absence of symptomatic cases. While ES has mainly been implemented in OPV countries that are considered as at-risk of VDPV emergences [26], ES constitutes a powerful tool that is also relevant in IPV countries to detect importation and silent circulation of PVs.

## Conclusion

The detection of cVDPV3s in French Guiana is a reminder that no territory is immune to PV importation as long as wild and vaccine PV strains are circulating. Maintaining PV surveillance systems is crucial to allow early detection of importation. For this purpose, ES constitutes an important tool for the Global Polio Eradication Initiative by enabling the detection of silent PV circulation.
